# Decreasing the options’ number in multiple choice questions in the assessment of senior medical students and its effect on exam psychometrics and distractors’ function

**DOI:** 10.1186/s12909-023-04206-3

**Published:** 2023-04-05

**Authors:** Manar Al-lawama, Ben Kumwenda

**Affiliations:** 1 School of Medicine, The University of Jordan, Amman, Jordan; 2 School of Medicine, Dundee University, Dundee, Scotland, UK

**Keywords:** Distractors, Exam psychometrics, MCQ, Number of options

## Abstract

**Background:**

Studies that have investigated the effect options’ number in MCQ tests used in the assessments of senior medical students are scarce. This study aims to compare exam psychometrics between three- and five-option MCQ tests in final-year assessments.

**Methods:**

A cluster randomized study was applied. Participants were classified into three groups, according to their academic levels. Students in each of those levels were randomized into either the three- or five-option test groups.

**Results:**

Mean time to finish the five-option test was 45 min, versus 32 min for the three-option group. Cronbach’s alpha was 0.89 for the three-option group, versus 0.81 for the five-options, *p*-value = 0.19. The mean difficulty index for the three-option group was 0.75, compared to 0.73 for the five-option group, *p*-value = 0.57. The mean discriminating index was 0.53 for the three-option group, and 0.45 for the five-options, *p*-value = 0.07. The frequency of non-functioning distractors was higher in the five-option test, 111 (56%), versus 39 (39%) in the three-options, with *p-*value < 0.01.

**Conclusions:**

This study has shown that three-option MCQs are comparable to five-option MCQs, in terms of exam psychometrics. Three-option MCQs are superior to five-option tests regarding distractors’ effectiveness and saving administrative time.

## Background

Globally, multiple choice question (MCQ) tests remain one of the most efficient, cost effective, and common methods of assessment. MCQs contain a stem and a number of options from which the candidate can choose the single best answer. Despite their popularity, the construction guidelines [1, 2] and item analysis guides [3] of MCQs remain a subject of debate. One of the debatable issues that is rarely explored in medical education is the optimal number of options that should be used in an MCQ test.

In recent years, educators have sought to improve the reliability and validity of MCQs [4]. Most institutions use four- or five-option MCQ tests [5]. Using four or five options is justified as being better able to maintain the reliability and validity of the assessment by decreasing the rate of random guessing [6]. By contrast, there is an emerging school of thought that advocates for three-option MCQs. Since then, there have been studies demonstrating that well-constructed three-option MCQs have better psychometric parameters; this is partially due to fewer non-functioning distractors (NFDs) [7, 8].

Moreover, the adoption of three-option MCQ tests can potentially increase exam efficiency by decreasing the time required for preparation and administration of the exam [9–11].

Studies that investigate the plausibility of three-option MCQs in medical education are scarce, especially in the final years of student instruction. Therefore, this study aims to contribute to the evidence regarding the optimal number of options in MCQ tests administered during students’ clinical years.

## Methods

We investigated whether using a three-option MCQ test, in comparison with a five-option MCQ test, affects an exam’s psychometric parameters. This was an interventional randomized study involving two homogenous groups of students who answered the same sets of questions with a different number of options. The first group was assigned to the three-option MCQ exam, while the second group sat the five-option MCQ. We compared the exams’ reliability, difficulty, discrimination ability, and frequency of NFDs.

### Research setting and participant recruitment

The study was conducted at the University of Jordan. The university runs a 6-year MD program into which students are accepted directly after finishing high school. The clinical years are the last 3 years of study. Students’ summative assessment consists of an end-of-rotation objective structured clinical examination and a written MCQ test at the end of the year.

Students eligible to participate in this study were those in their final year who had completed 16 weeks of training in pediatrics. Due to the COVID-19 pandemic and related restrictions, the decision was made to administer the exams online. The study was approved by the University of Jordan and the University of Dundee ethics committees.

### Randomization

The cluster randomization method was used. The students who agreed to participate were sorted into three groups according to their pre-test GPAs: high performance, intermediate performance, and low performance. To ensure a similar distribution of students meeting these performance levels in both study groups, the students in each of the clusters were randomized into either the five-option MCQ group (control group) or the three-option MCQ group (experimental group).

### MCQs preparation for both test versions

Fifty MCQs were chosen from previously validated and administered tests. These 50 MCQs with five options formed the “traditional test”, which was assigned to the control group. The options were reviewed by another faculty member, in addition to the first author. Both faculty members agreed on the two options that were expected to be the least attractive to the students, and they were eliminated to form the three-option test assigned to the experimental group.

All items were presented in English and evenly distributed to cover the taught curriculum. The items were a blueprint of the school’s summative assessment. The items were variable in terms of their length, structure, and cognitive level and were distributed in a balanced way among cognitive levels according to Bloom’s taxonomy [12]. For example, 25 items (50%) covered application and critical thinking, and another 25 encompassed recall and comprehension. The items were divided into two types according to what they measured. The first type was targeted skill questions, in which the students were asked to choose the best type of investigation, the most likely diagnosis, or the best treatment approach (Table 1). The second type included questions that inquired about general disease knowledge (Table 2).

**Table 1:** Targeted skill item in its 5- and 3-option formats

Five-Option Format	Three-Option Format
Q. A 12-year-old girl presented with several syncopal episodes over the past 8 months; there was an associated feeling of chest discomfort with a strong heartbeat prior to the syncopal attacks. On examination, her blood pressure and heart rate are normal, and there is a prominent right ventricular impulse with a loud second heart sound. An ECG shows right ventricular hypertrophy. The most likely diagnosis in this girl is	Q. A 12-year-old girl presented with several syncopal episodes over the past 8 months; there was an associated feeling of chest discomfort with a strong heartbeat prior to the syncopal attacks. On examination, her blood pressure and heart rate are normal, and there is a prominent right ventricular impulse with a loud second heart sound. An ECG shows right ventricular hypertrophy. The most likely diagnosis in this girl is
*A-Vasovagal syncope* *B-Pulmonary hypertension* *C-Anemia due to heavy menstruation* *D-Coarctation of aorta* *E-Long QT syndrome* *Key: B*	*A-Vasovagal syncope* *B-Pulmonary hypertension* *C-Long QT syndrome* *Key: B*

**Table 2:** General disease knowledge item in its 5- & 3-option formats

Five-Option Format	Three-Option Format
Q. One of the following lab abnormalities is expected in the case of chronic liver disease: *A-High ALT* *B-Low Gamma GT* *C-High INR* *D-Normal Albumin* *E-High AST* *Key: C*	Q. One of the following lab abnormalities is expected in the case of chronic liver disease: *A-Low Gamma GT* *B-High INR* *C-Normal Albumin* *Key: B*

Thirty-five items (70%) targeted specific clinical skills. The remaining 15 items (30%) measured general disease knowledge. There were 30 (60%) scenario-based items. Twenty-nine items (58%) had a stem length of more than 20 words and were designated as long-stem items.

### Time allocation

The five-option test was allotted the completion time recommended by the school guide, 90 s per item. To calculate the time for the three-option format, we first determined the assumed time needed for each option in the five-option format based on the following calculation: 50 s were arbitrarily given to read the stem, and 40 s were left to be shared among the five options, resulting in 8 s for each option. Therefore, each item in the three-option test was allocated roughly 16 s less, shortening the whole test by about 10 min. Since our traditional test is paper-based and includes time for completing the answer sheet.

### Test administration

After randomization, two WhatsApp® groups were created. The students were contacted by sending a welcome message and test instructions. The test was done on a Friday afternoon which was lockdown day due to COVID-19 situation. Google Forms were used to create the test. The test started by thanking the students for their participation and explaining its nature, including their anonymity. Question number one asked the participants to confirm their consent. Then, the test MCQs started with question number two. The test was real-time. The test link was sent on the WhatsApp® group 2 min prior to the exam start time. The link was set to open at 5:00 pm Amman local time and closes when the test time is finished; 60 min for the three-option group and 70 min for the five–option group. It was not proctored.

### Ethical considerations

We made direct contact with the students through WhatsApp groups and shared the study information with them. The Google Form started by asking the student if he or she agreed to participate or not. Those who agreed to participate submitted the test forms.

The tests were gathered anonymously. The Google Forms were set so that the students only needed a link to access them, eliminating the need to register their email addresses. The forms did not require the students to provide their name or university ID number. All forms were stored securely using password protection, and only the researcher had access to them.

### Statistical analysis

All test answers were downloaded into Microsoft Excel for analysis. We calculated the mean, standard deviation (SD), difficulty index (DF), discrimination index (DS), reliability, standard error of measurement (SEM), and frequency of NFDs from both groups. The detailed statistical methods are discussed in each relevant section below.

### The difficulty index

The difficulty index (DF) is the percentage of examinees who answered an item correctly. It is calculated by dividing the number of students who answered the item correctly (C) by the total number of students (T), C/T = DF. The larger the number, the easier the item. The DF can range from 0 to 1. The higher the number, the easier the item [13]. The desirable difficulty level is slightly higher than midway between chance and a perfect score for the item. Thus, for five-option and three-option MCQs, the recommended DFs are 0.70, and 0.77, respectively [14].

For this study, the items were classified as easy, acceptable, or difficult, according to their difficulty levels. An easy item is one where 80% or more of the students chose the correct response. When more than 30%, but less than 80%, of the students chose the correct response, the difficulty level was acceptable. If 30% or less of the students chose the correct response, the item was classified as difficult [15].

### The discrimination index

The discrimination index (DS) reflects the ability of an item to discriminate between a high-scoring examinee and a low achiever on a test [16]. The DS ranges from -1 to 1. The larger the value of the DS, the more discriminating the item. Negative discrimination items mean that more students in the low-performing group were able to answer the item correctly than students who performed highly. These items are usually faulty ones [17]. The DS was calculated by subtracting the proportion of low-performing students who answered the item correctly (L) from the proportion of high-performing students who answered the item correctly (U), U-L = DS.

To calculate the DS in this study, the scores for each test version were arranged in descending order. Then, the upper and lower 27% of the students’ scores were identified. An item’s discrimination ability was classified as excellent if the DS was > 0.4, and good, acceptable, or poor if it was within the range of 0.3–0.39, 0.2–0.29, or 0–0.19, respectively [18].

### Reliability

Reliability in measurement reflects the concordance of the “observed score” with the “true” score, which is measured with no error. Cronbach’s alpha (α) is a test of internal consistency [19]. It was used in this study to reflect reliability. A reliability coefficient α of 0.8 or more is desired for high-stakes, in-house exams [20, 21]. It was calculated using the following formula:

$$\mathit(\alpha\mathit)\mathit=Q\mathit/\mathit(Q\mathit-\mathit1\mathit)\mathit\ast\mathit(\mathit1\mathit-\mathit(Sum\mathit\;of\mathit\;variances\mathit\;of\mathit\;allitems\mathit/total\mathit\;test\mathit\;variance\mathit)\mathit)$$

where *Q* is the total number of items.

Statistical comparisons were made between Cronbach’s alpha coefficients using an online calculation tool [22]. Calculations rely on the tests implemented in the “cocron” package for the R programming language [23].

### Standard error of measurement (SEM)

The standard error of measurement (SEM) measures to what extent test scores are spread around a “true” score [24]. It was calculated using the following formula:

$$\mathit(SEM\mathit)\mathit=SD\mathit\ast\mathit\surd\mathit(\mathit1\mathit-reliability\mathit).$$

### Non-functioning distractors (NFDs)

For this study, a non-functional distractor (NFD) is defined as a distractor chosen by < 0.05 of the students [10]. The correlation between the frequency of NFDs and both exam difficulty and discrimination ability were calculated using Pearson’s correlation coefficient (*r*). If *r* is between 0 and 0.19, the association is regarded as very weak, 0.2–0.39 indicates it is weak, 0.40–0.59 shows it is moderate, 0.6–0.79 reflects it as strong, and 0.8–1 indicates a very strong correlation [25]. An online tool was used to calculate the Pearson’s coefficient and its significance [26].

## Results

### Participants’ characteristics

**Fig. 1:**
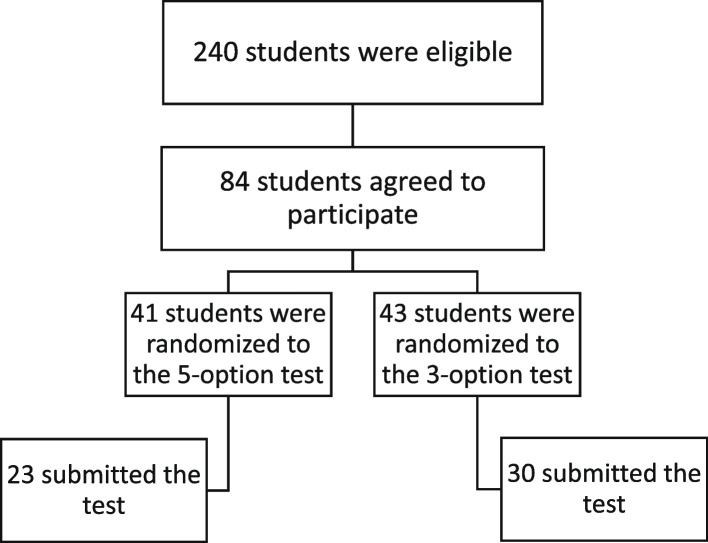
Recruitment & Randomization of The Study Participants

A total of 240 students met the inclusion criteria. Of these, 84 agreed to participate. Forty-three were randomly assigned to the five-option test, and of these, only 30 submitted the completed test results. Forty-one students were randomly assigned to the three-option test, and of these, only 23 completed the test. Figure 1 shows the flowchart of the participants’ recruitment.

There was an equal gender distribution between the two study groups. Most of the included students were female; this reflects the gender distribution for this class, where females constitute 58% of the students. Most of the participants had an intermediate GPA (Table 3).

**Table 3:** Participants’ characteristics

Characteristic	3-Option Group (41)	5-Option Group (43)	*P*-Value
Gender			
Female	25 (61%)	26 (60%)	1.00
Male	16 (39%)	17 (40%)	
Pre-Test GPA			
Low GPA	17 (41%)	18 (42%)	1.00
Intermediate GPA	20 (49%)	21 (49%)	
High GPA	4 (10%)	4 (9%)	

### Test score analyses

The maximum possible score on both tests was 50. The mean ± SD for the five-option test was 36.53 ± 6.44, with a score range of 20–44. The equivalent for the three-option test was 37.74 ± 7.85, with a score range of 14–47. This difference was not statistically significant (*p-*value = 0.54). The passing score was 25 (50%) for both exams. Six percent of the students in the five-option test failed the exam, compared to 4% in the three-option test.

### Reliability and the standard error of measurement

The three-option test was more reliable than the five-option one, with reliability coefficients of 0.89 and 0.81, respectively, *p-*value = 0.19. The reliability was also compared among all the MCQ categories (Table 4). The SEM was lower for the three-option test (2.6) than for the five-option one (2.8).

**Table 4:** Reliability of three options and five options categories

Category	Number of Items	Three Options	Five Options	*P*-Value
All	50	0.89	0.81	0.19
Application/Critical Thinking	29	0.84	0.80	0.59
Recall/Comprehension	21	0.70	0.35	0.07
Long Stem	29	0.82	0.80	0.80
Short Stem	21	0.75	0.27	0.01
Targeted Skill	35	0.86	0.82	0.54
General Disease Knowledge	15	0.53	0.17	0.19

### Difficulty index

The three-option test was easier, as the mean DF for all items was higher for three-option format: 0.75 ± 0.18 and 0.73 ± 0.17, while for the five-option test, the *p-*value was 0.57. The DF was also calculated according to the item categories. The three-option test’s DF was equal to or higher than the five-option test’s DF in all categories (Table 5).

**Table 5:** Difficulty index comparison according to items’ category

Domain	Five Options (DF)	Three Options (DF)	*P*-Value
All Items	0.73 ± 0.17	0.75 ± 0.18	0.57
Recall/Comprehension	0.68 ± 0.16	0.72 ± 0.19	0.42
Application/Critical Thinking	0.77 ± 0.17	0.77 ± 0.17	1.00
Targeted Skill	0.76 ± 0.17	0.77 ± 0.16	0.80
General Disease Knowledge	0.65 ± 0.16	0.68 ± 0.22	0.67
Long Stem	0.75 ± 0.16	0.75 ± 0.18	1.00
Short Stem	0.70 ± 0.18	0.74 ± 0.19	0.49

**Fig. 2:**
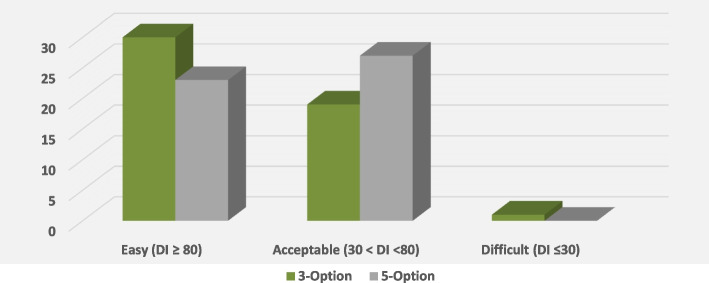
Items’ Distribution According to Difficulty Indices

Most items in the three-option format were found to be easy, 30 (60%), while in the five-option format, most of the items had an acceptable difficulty level, 27 (54%) (Fig. 2).

### Discrimination index (DS)

The mean discrimination index (DS) was 0.53 for the three-option items and 0.45 for the five-option items. The three-option items’ DS was higher in all items’ categories (Table 6).

**Table 6:** Discrimination index comparison according to items’ category

Domain	Five Options (DS)	Three Options (DS)	*P*-Value
All Items	0.45 ± 0.21	0.53 ± 0.22	0.07
Recall/Comprehension	0.45 ± 0.20	0.53 ± 0.25	0.20
Application/Critical Thinking	0.46 ± 0.23	0.53 ± 0.19	0.25
Targeted Skill	0.45 ± 0.20	0.53 ± 0.19	0.09
General Disease Knowledge	0.47 ± 0.24	0.53 ± 0.28	0.53
Long Stem	0.46 ± 0.21	0.53 ± 0.20	0.20
Short Stem	0.48 ± 0.22	0.52 ± 0.24	0.58

**Fig. 3:**
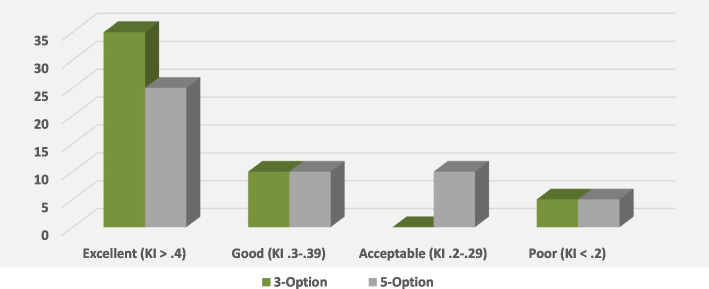
Items’ Distribution According to Discrimination Indices

Most of the items in both versions had excellent discrimination; however, there were more items with excellent discrimination in the three-option format (70 vs. 50%, Fig. 3).

### Distractors’ performance

We analyzed 300 distractors: 200 in the five-option test and 100 in the three-option test. The frequency of NFDs (those chosen by < 0.05 of the students) was higher in the five-option test: 111 NFDs (56%), versus 39 (39%) in the three-option test, *p-*value < 0.01. Of these NFDs, 73 (66%) were chosen by no students from the five-option group, versus 15 (38%) from the three-option group, *p-*value < 0.01. In the five-option test, 80% of the items had two or more NFDs. In the three-option format, 36% had no NFDs, while in the five-option format, only 6% had no NFDs (Table 7).

**Table 7:** Comparison of NFDs frequency in the current study

NFDs per Item	Three Options	Five Options	*P*-Value
0	18 (36%)	3 (6%)	0.00
1	20 (40%)	7 (14%)	0.00
2	12 (24%)	18 (36%)	0.27
3	NA	13 (26%)	NA
4	NA	9 (18%)	NA

The performance of each NFD in each version was compared with its performance in the other version. Of the 111 NFDs in the five-option test, 29 performed poorly in the three-option test. Of these, 19 had variable levels of performance, and 80% were chosen by < 0.09 of the students, ranging from 8 to 34%. The remaining 63 (57%) were expected to be non-plausible and therefore deleted when the three-option test was created. A performance comparison of the same distractor in both test versions could not be conducted.

**Fig. 4:**
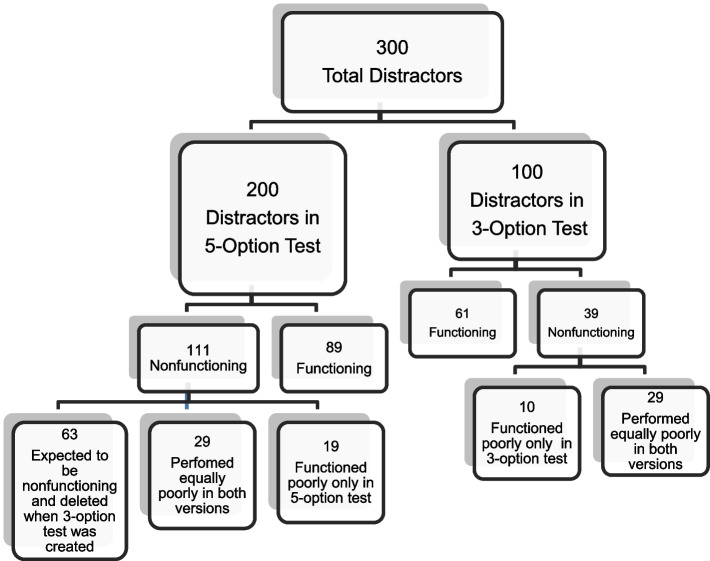
Distribution of The NFDs Between Test Versions

Of the 39 NFDs in the three-option test, 29 performed poorly in the five-option test. The remaining 10 had variable performance, ranging from 8 to 34%. Of these, 80% were chosen by < 0.07 of the students (Fig. 4).

### Correlation between NFDs and items’ DF and DS

There was a positive correlation, *r* = 0.71 and 0.81, for the three and five option tests, respectively, *p-*value < 0.01, for both versions. As for the correlation with an item’s discrimination ability, it was negative: *r* = -0.44, *p-*value < 0.01 for the three-option test and *r* = -0.38, *p-*value < 0.01 for the five-option version.

### Test time

The mean time students needed to finish the five-option test was 45 min and 49 s, while it was 32 min and 58 s for the three-option test. This means they required 55 s per item to consider the five options and 40 s per item to consider the three options. The difference was 15 s per item. This results in an estimated 7 s per option.

## Discussion

The findings of this study join the conversation on the feasibility of three-option MCQs following a reduction from five options. The study has demonstrated that a well-constructed three-option MCQ can significantly reduce the time required to administer the exam without losing the psychometric properties of the assessment tool. Both test versions had a high level of reliability, with Cronbach’s alphas of 0.89 and 0.81 for the three- and five-option tests, respectively (Table 4).

Most studies that compare the impact of the number of options on an exam analysis did not investigate reliability [9]. Few of the studies that have looked at the reduction of MCQ options from four to three have reported that there was no noticeable change in the reliability of the exams [8, 10, 27, 28]. Very few studies have compared the reliability of three and five options, but they, too, found no significant differences [29, 30].

This study also compared the reliability of a three-option MCQ according to question categories: the cognitive levels targeted, question length, and structure. There was a consistent pattern of higher reliability for the three-option test in all categories, compared to the five-option test. Budescu and Nevo [31] investigated the effect of question complexity on the reliability difference between three and five options and found that the relationship between the number of options and reliability varied according to the skill being tested, and whether it was related to vocabulary, reading comprehension, or mathematics. They called this variability the “m” factor, which reflects the question’s complexity. When the complexity approached zero, the three-option test yielded maximum reliability, evident in its short vocabulary questions. However, in more complex questions, five options provided higher reliability.

In this study, the SEM was slightly lower for the three-option test: 2.6, versus 2.8 for the five-option test. This means that we are 95% confident that the true score of any student on the three-option test lies within 5.2 points of the student’s exam score, versus 5.6 for the five -option test [16]. The SEM is strongly related to reliability. The larger the test reliability estimate, the lower the SEM. However, the SEM helps us evaluate a particular student’s score. To the best of our knowledge, only one study to date has reported on the use of the SEM to investigate the optimum number of MCQ options, and it compared three and four options [27]. That study’s findings were consistent with ours, where we found a slight decrease in the SEM for the three-option format.

### Difficulty and discrimination ability

The mean scores and standard deviation for the three-option test were higher than those for the five-option test: 37.74 ± 7.85, versus 36.53 ± 6.44, respectively. However, this difference is not significant, *p* = *0.54.* This is consistent with Tarrant and Ware’s [28] findings when comparing three and four options in nursing student assessments. However, Rogers and Harley [27] found that the mean score of a three-option test was significantly higher than that of a four-option one: 20.42 ± 4.88, versus 17.81 ± 4.61. However, they included high school students, and the test was on mathematics.

Most of the items in the three-option test were considered easy, while they were mostly of an acceptable level in the five-option format (Fig. 2). This pattern continued upon computing the item DF, which showed that the three-option format was slightly easier than the five-option format: 0.75 ± 0.18, versus 0.7 ± 0.17. However, this was not significant, *p* = 0.57. This finding is consistent for all the MCQ categories (Table 5). The students who participated in this study were in their final year of medical school and were expected to perform at this level of proficiency in the exam subject.

Item difficulty is the most-studied parameter regarding the optimum number of options in MCQs [9]. The findings of the present study are consistent with a meta-analysis performed by Rodriguez [10]. He found that all the included studies he reviewed showed that a reduction in the number of options was always associated with an increase in DF, making the fewer-option format easier. However, the most significant effect was observed upon decreasing the option number to two. The meta-analysis results are also consistent with many not included and later studies [7, 8, 27]. To our knowledge, one study, by Tarrant and Ware [27], found that three-option tests were more difficult than four-option formats, with DF of 0.70 ± 0.5, versus 0.73 ± 0.14, respectively, yet their results were not significant. Many factors might have contributed to this different finding. For example, Tarrant and Ware administered the two tests in two different years with different numbers of items. Furthermore, a considerable number of the items were not common between the two versions.

In this study, most of the items in the three-option test showed excellent discrimination ability: DS ≥ 0.4 with a frequency of 70%, versus 50% for the five-option test. The mean DS was higher for three options: 0.53, versus 0.45 for five options. This was consistent for all the MCQ categories (Fig. 3). However, this finding was not significant. The DS reflects a question’s quality and how it can differentiate among students’ abilities [32]. This element is less studied than the DF when investigating the optimum number of options [9]. In Rodriguez’s meta-analysis [10], studies that investigated the effect on the DS showed that decreasing the number of options is associated with a reduction in items’ discrimination ability. This reduction was observed to be the smallest when changing from five to three options. However, Rodriguez included studies from all fields of science and different educational levels. Many other studies have shown no difference in the DS when changing the number of options [8, 27, 33, 34].

### Distractors’ performance

The most frequent pattern of NFDs in the five-option format was two per item (36%), while only 6% had four functioning distractors (Table 7). This high rate of NFDs is consistent with a study conducted by Kilgour and Tayyaba [35]. They reviewed four MCQ tests with five-option questions designed for medical students and found that 33.1% had two functioning distractors, ranging from 30.5% to 39.3%, and that only 7.1% had four functioning distractors, ranging from 5.5% to 8.6%. Fozzard et al. [36] reviewed an assessment administered to medical students and found that 26% had two functioning distractors, ranging from 20 to 33%, while 19% of MCQs had four functioning distractors, ranging from 4 to 28%.

The overall frequency of NFDs in the five-option test was higher than that in the three-option test: 56%, versus 44%, respectively, *p-*value = 0.00. Items with 100% effective distractors (0 NFDs) were significantly higher for the three options: 36%, versus 6% for the five options, *p-*value = 0.00. Items with zero functioning distractors (100% NFDs) comprised 24%, versus 18% in the three- and five-option tests, respectively, but this result was not statistically significant, *p-*value = 0.27.

This finding is consistent with the published literature, where more options have been found to be associated with more NFDs [8, 9, 34]. This finding of such a high rate of NFDs in the five-option test, and the significant reduction in NFD frequency that occurs with decreasing the number of options, further supports the assumption by Haladyna and Downing [9] that three options per item may be a natural limit for MCQ item-writers in most circumstances. For any problem that is presented to students, there are a limited number of plausible solutions. When the number of options is predetermined, as per the test regulation, item-writers might fulfil the requirement by using poorly constructed, implausible options.

Upon further analysis of distractor performance and its relation to an item’s difficulty and discrimination ability, there was a strong positive correlation between the number of NFDs per item and the item’s DF, where the more NFDs, the easier the item: *r* = 0.71 and 0.81 for three and five options, respectively, *p-*value < 0.01 for both. There was a moderate negative correlation between the frequency of NFDs per item and the item’s discrimination ability for both test versions: *r* = *-*0.44 and -0.38 for the three and five options, respectively, *p-*value = 0.00*.* This is consistent with previous studies [37, 38]. This finding is essential for further proving the importance of designing effective and plausible distractors. In this study, the three-option test showed a higher discrimination ability than the five-option test, a finding that can be attributed to the fact that the three-option test had more functioning distractors than the five-option format.

This study also showed that 53% of the NFDs in the five-option test were expected to perform poorly by experts and deleted when the three-option test was created (Fig. 4). This observation further supports previous studies that found that experts were able to detect NFDs without conducting a formal exam analysis [39–41]. The other important observation regarding distractor performance is that 29 distractors performed equally poorly in both test versions and were NFDs (Fig. 4). These two observations should draw the exam constructor’s attention to the idea that a distractor’s plausibility is an inherent quality and not related to the number of options. Therefore, training on item-writing should improve the performance of distractors and, potentially, positively impact reliability and discrimination ability [41].

### Test time

The time calculated before the administration of the test followed many theoretical and empirical studies that claim that the test’s allotted time should be proportional to the test’s length [42, 43]. Based on this principle, eight seconds were allowed per option; therefore, it was estimated that 10 min less time was required for the three-option test. This is the first study to consider a different test time for each option-number version. All previous studies calculated time as an outcome [31, 33, 34, 42, 43]. The actual time that the students took to finish each test was 10 min less than the pre-administration calculation. The difference between the two tests was 15 s per item. Since the items were identical, one can assume the difference was due to the distinct numbers of options, meaning that each option needed 7 s. This study’s findings reveal that the time difference between the two versions per item is greater than previously reported. Vegada et al. [34] reported a savings of 6 s per item, in comparison to 15 s in our study. This might partially be explained by the fact that they used a more difficult test than we did.

## Conclusions

This study adds to the current literature that claims the optimum number of options in MCQ tests is three. Concerns about the reliability of this type of exam are not supported by evidence. Most educators who argue against using three-option formats base their opinion on the chance of guessing the correct response [44], which increases from 20% in the five-option format to 33% when there are only three options. Even though this is only a theoretical argument and not supported by accumulated evidence over the last 100 years, it is based on applying principles of random guessing. The measurement of students’ knowledge of a taught curriculum using MCQ questions is not based on sheer guessing, as Costin [45] realized more than 45 years ago. Later, this theory was supported by many other studies [10, 31]. The principle of random guessing is applied if all the options have an equal probability of being chosen; however, tests are usually designed to meet the learning objectives of a taught curriculum, and test takers are expected to have full or partial knowledge of the test items, so they approach each option with some degree of knowledge, which is certainly not random guessing [46]. Examinees are familiar with the exam subject. Hence, they can narrow down the possible correct responses and change the provided number of options from five to a lower number of options according to their competency in that subject [30, 47].

Based on this study and the medical literature, we recommend the adoption of three-option MCQs. However, while making this recommendation, we also emphasize that the goal is not simply to have fewer distractors, but fewer well-functioning distractors [7]. In addition, and contrary to most previous studies that have recommended adopting three-option MCQs, we would like to propose a more individualized approach that depends on the evaluation of each medical school’s MCQ-based assessments. Each school should perform an extensive review of its MCQ tests and evaluate the quality of their items. They should calculate the exams’ reliability and assess the frequency of NFDs in their test items. Subsequently, they should decide if switching to a three-option format would improve their exams’ psychometrics.

This study has proved that three-option MCQs are comparable to five-option MCQs, in terms of exam psychometrics. Three-option MCQs are superior to five-option tests with regard to saving time in relation to their construction and administration, and they allow for the better presentation of a curriculum. The choice of adopting a specific MCQ format should depend on each school’s assessment situation and the quality of its MCQs. Future research should focus on improving the quality of MCQ tests and on designing and implementing guidelines on MCQ construction, especially those related to creating plausible distractors.

## Data Availability

The datasets used and/or analysed during the current study are available from the corresponding author on reasonable request.
